# Flavonoids as a Natural Enhancer of Neuroplasticity—An Overview of the Mechanism of Neurorestorative Action

**DOI:** 10.3390/antiox9111035

**Published:** 2020-10-23

**Authors:** Natalia Cichon, Joanna Saluk-Bijak, Leslaw Gorniak, Lukasz Przyslo, Michal Bijak

**Affiliations:** 1 Biohazard Prevention Center, Faculty of Biology and Environmental Protection, University of Lodz, Pomorska 141/143, 90-236 Lodz, Poland; leslaw.gorniak@biol.uni.lodz.pl (L.G.); michal.bijak@biol.uni.lodz.pl (M.B.); 2 Department of General Biochemistry, Faculty of Biology and Environmental Protection, University of Lodz, Pomorska 141/143, 90-236 Lodz, Poland; joanna.saluk@biol.uni.lodz.pl; 3 Department of Developmental Neurology and Epileptology, Research Institute of Polish Mother’s Memorial Hospital, Rzgowska 281/289, 93-338 Lodz, Poland; lukasz.przyslo@iczmp.edu.pl

**Keywords:** flavonoids, neuroplasticity, synaptogenesis, neurogenesis

## Abstract

Neuroplasticity is a complex physiological process occurring in the brain for its entire life. However, it is of particular importance in the case of central nervous system (CNS) disorders. Neurological recovery largely depends on the ability to reestablish the structural and functional organization of neurovascular networks, which must be pharmacologically supported. For this reason, new forms of therapy are constantly being sought. Including adjuvant therapies in standard treatment may support the enhancement of repair processes and restore impaired brain functions. The common hallmark of nerve tissue damage is increased by oxidative stress and inflammation. Thus, the studies on flavonoids with strong antioxidant and anti-inflammatory properties as a potential application in neuro intervention have been carried out for a long time. However, recent results have revealed another important property of these compounds in CNS therapy. Flavonoids possess neuroprotective activity, and promote synaptogenesis and neurogenesis, by, among other means, inhibiting oxidative stress and neuroinflammation. This paper presents an overview of the latest knowledge on the impact of flavonoids on the plasticity processes of the brain, taking into account the molecular basis of their activity.

## 1. Introduction

Civilizational development, advances in medicine and lifestyle changes, including dietary changes, contribute to the constant improvement of the quality of human life. On the other hand, the growing number of seniors results in an increasing percentage of the population suffering from miscellaneous central nervous system (CNS) diseases. Despite significant progress being made in neurobiology over recent years, it is still not possible to precisely define the mechanism leading to nervous tissue pathology. Serious difficulties in developing effective drugs result from limited bioavailability, depending on their ability to cross the blood–brain barrier (BBB), as well as their rapid degradation, leading to the need to administer high concentrations of substances, which indicates the need to search for new therapeutic solutions.

The regenerative processes of the brain tissue are characterized by a limited scope and are regulated by the properties of the tissue environment, dependent on changes in the physiology of the body. The brain’s ability to create structural and functional adaptive changes is known as brain plasticity. This process includes neurogenesis, synaptogenesis, and neurochemical changes of the CNS. Therefore, the regulation of neuroplasticity processes raises high clinical hopes, and its forms include pharmacotherapy and biological therapy, causing a synergistic effect between neurogenesis and synaptogenesis [1]. Physical activity, including properly conducted rehabilitation, is an important treatment option inducing neurogenesis, strongly correlating with the improvement of the memory and attention of patients. High-intensity exercise has been shown to increase neurogenesis in the hippocampus, while medium and low-intensity exercise improves the maturation and survival of newly formed neurons [2]. Moreover, the plasticity processes are also influenced by therapeutic methods based on physical stimuli, e.g., therapeutic hypothermia, deep brain stimulation (DBS) and transcranial magnetic stimulation (TMS) [3].

The compensatory plasticity occurring in a damaged brain is an entirely different process than plasticity in a healthy brain. This process begins in critical conditions related to inflammation, edema, the degeneration of nerve fibers, apoptosis and metabolic disorders. Synaptogenesis is based on enhancing existing synaptic pathways, and then forming new connections. The remaining but diminished boutons are activated, other subcortical or cortical structures take over the function of the impaired brain region, and as a result, the functions of the damaged area may be restored [4]. 

**Figure 1:**
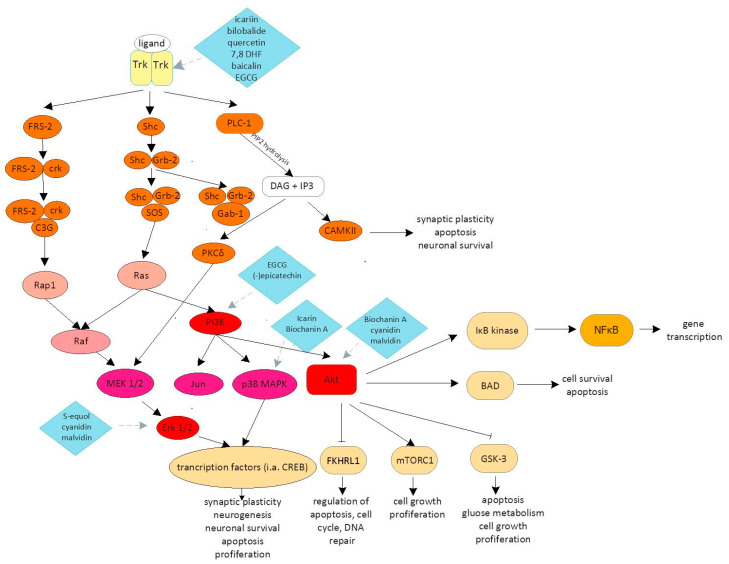
Schematic diagram of the signaling cascades most important in the neuroprotection, proliferation and survival of neurons, including the phospholipase C/inositol trisphosphate/Ca^2+^/calmodulin-dependent protein kinase II (PLC/IP3/CAMKII), phosphoinositide-3-kinase–protein kinase B/protein kinase B (PI3K/Akt) and mitogen-activated protein kinase/extracellular signal-regulated kinase 1/2 (MAPK/Erk) pathways. The activation of these pathways leads to increased neuronal outgrowth, the inhibition of apoptosis, enhanced synaptogenesis, as well as increased generation of growth factors dependent on neurotrophic factors. Potential sites of flavonoid interference have been marked on individual signaling pathways. A detailed description with an extension of the abbreviation is included in the text.

The most important signaling pathways involved in brain plasticity include the phosphoinositide-3-kinase–protein kinase B/protein kinase B (PI3K/Akt), phospholipase C/inositol trisphosphate/Ca^2+^/calmodulin-dependent protein kinase II (PLC/IP3/CAMKII), and mitogen-activated protein kinase/extracellular signal-regulated kinase 1/2 (MAPK/Erk) pathways, which are activated by multiple growth factors: brain-derived neurotrophic factor (BDNF), insulin-like growth factor 1 (IGF1), nerve growth factor (NGF), fibroblast growth factor (FGF), and Wnt (Figure 1) [5]. 

Neurotrophic factors bind to two classes of receptor: tropomyosin receptor kinase (Trk), belonging to the family of tyrosine kinase receptors, and the p75 receptor, a member of the tumor necrosis factor receptor superfamily (due to it possessing a cytoplasmic “death” domain structurally similar to those in other members of this receptor family). Through these, neurotrophins regulate the survival of neurons and ensure their proper development and normal neuronal function. For many years after its discovery, the p75 receptor was believed to be only a binding protein or a low-affinity receptor, only specific for NGF. Unlike the receptors that autophosphorylate after ligand engagement, p75 does not contain a catalytic domain to autoactivate the receptor. Devoid of catalytic activity, p75 functions largely via interactions with other effector proteins. While a great deal remains to be discovered about the wide-ranging functions of p75, it is well documented that it interacts with and modulates the functions of TrkA, TrkB and TrkC receptors [6]. Trk receptors are composed of two immunoglobin-like domains responsible for ligand binding, the transmembrane domain and the cytoplasmic domain containing the tyrosine kinase domain. The direct binding of growth factors to Trk receptors causes their dimerization and phosphorylation of the cytoplasmic domain, leading to the activation of tyrosine kinases [7]. The activation of signaling pathways through Trk receptors requires adapter proteins containing Src Homology 2 (SH-2) or phosphotyrosine-binding (PTB) domains, which then induce intracellular signaling PI3K/Akt pathways leading to the activation of the expression of genes involved in brain plasticity. In the cytoplasmic domain, Trk receptors contain 10 highly conserved tyrosine residues, 3 of which (Y670, Y674 and Y675) are responsible for the control of enzymatic activity, while the phosphorylation of the remaining tyrosine residues promotes the formation of adapter protein docking sites [8]. The phosphorylation of the Trk receptor causes the activation of PLC-1, which in turn catalyzes the hydrolysis of phosphatidylinositol 4-,5-bisphosphate (PIP2), leading to the formation of the next transmitters: diacylglycerol (DAG) and inositol triphosphate (IP3). IP3 induces the release of Ca^2+^ stores and thus increases the level of cytoplasmic Ca^2+^. The attachment of four calcium ions activates the calmodulin modulator protein, which changes its conformation. In this form, it stimulates the action of many enzymes, including CaMK and calmodulin-dependent fosfatase, while the production of DAG stimulates the activity of DAG-dependent protein kinase C isoforms (PKC*δ*), which induces the MAPK/Erk pathway [9]. Ras protein plays an important role in the brain plasticity and activates the MAPK/Erk or PI3K/Akt pathway. Activation of the MAPK/Erk pathway leads to the transcription of protein factors involved in synaptogenesis and neuritogenesis, including cAMP response element-binding protein (CREB), Myc and ribosomal S6 kinase (RSK). The transient or prolonged activation of the MAPK/Erk pathway produces different molecular effects. The transient stimulation of MAPK/Erk is caused by the activation of Ras, which in turn is activated by the Src homology and containing protein (Shc)/growth factor receptor-bound protein 2 (Grb-2)/son of sevenless (SOS). Then, Erk kinase, through the RSK and MAP pathway, phosphorylates CREB and other transcription factors, leading to the regulation of the expression of genes responsible, inter alia, for the neurons’ survival. In contrast, the sustained activation of Erk involves the adapter protein fibroblast growth factor receptor substrate 2 (FRS-2) or Suc1-associated neurotrophic factor target (SNT). The phosphorylation of FRS-2 by Trk causes its association with adapter molecule crk, which in turn binds and activates the guanyl-nucleotide exchange (C3G) factor that stimulates the Ras-related protein 1 (Rap1). Protein Rap1 leads to the activation of the Erk kinase signaling pathway by binding to serine/threonine-protein kinase B-raf [10].

Brain plasticity processes are indissolubly associated with the activation of the PI3K pathway. The activation of PI3K results in the generation of phosphatidyl inositides responsible for the stimulation of protein kinase Akt/protein kinase B (PKB). Akt kinase plays an important role in controlling the functionality of many proteins involved in the regulation of cell survival. Among others, Akt, by phosphorylation of the Bcl2-associated agonist of cell death (BAD), inhibits apoptosis. In turn, the phosphorylation of nuclear factor kappa B inhibitor (IκB) by Akt leads to its degradation and NFκB stimulation, resulting in an increased survival of neurons. In addition, Akt limits apoptosis through the phosphorylation of the transcription factor forkhead 1 (FKHRL1), regulating the expression of apoptosis-promoting gene products such as ligand Fas (FasL), as well as by the negative regulation of glycogen synthase 3β kinase (GSK-3β), supporting apoptosis. Activation of PI3K is induced by Ras. Ras activation of PI3K is the most important signaling pathway responsible for the survival of neurons. In parts of the neurons, the Ras-dependent activation of PI3K is the major survival-promoting pathway, induced by neurotrophins. However, Ras-independent PI3K signaling may also occur. PI3K binds to the Grb2-associated-binding protein 1 (Gab-1) activated by phosphorylated Grb-2. The adapter protein Gab-1 and the formation of the complex facilitate the activation of PI-3 kinase [11].

Another important component of brain plasticity is neurogenesis, which is regulated by microenvironmental factors, neurotrophins, neurotransmitters, growth factors and hormones [12]. It has been shown that in certain brain regions, the differentiation of neuronal stem cells (NSCs) present in a fully developed brain into mature neurons occurs throughout life. However, the reduction in the rate of this process, as well as the survival capacity of the newly formed nerve cells, correlates with age. The strongest neurogenesis occurs mainly in the subgranular zone (SGZ) and the subventricular zone (SVZ), in the areas of the brain responsible for memory, learning and olfactory sensation [13].

Focusing the therapy on supporting neuroplastic processes seems to be a promising treatment strategy. One of its elements may be the use of natural substances that can be used both in neuroprotection and in supporting conventional treatment. 

Taking the above into account, the aim of this work is to review the latest research on the use of flavonoids as enhancers of neuroplasticity in the treatment of CNS diseases.

## 2. Flavonoids

**Figure 2:**
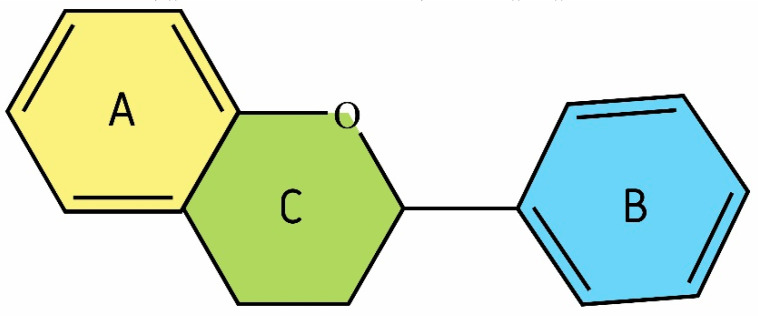
Structural characteristic of flavonoids; benzene rings A has been indicated in yellow, ring B in blue, pyron or pyran ring in green color (structure generated from InChI code available on https://pubchem.ncbi.nlm.nih.gov/) (accessed on 18 August 2020).

Due to their chemical structure, flavonoids (2-phenyl-benzo-γpyrone derivatives) are divided into flavanones, flavanols, flavones, isoflavones, flavonols, and anthocyanins. Flavonoid compounds also include biflavonoids, flavonolignans, prenylflavonoids, flavonoid glycosides, chalcones and proanthocyanins. Flavonoids contain a flavan backbone formed of two benzene rings (rings A and B) connected by a heterocyclic ring of pyron or pyran (ring C) (Figure 2). The classification of flavonoid compounds takes into account the presence of a carbonyl group at the fourth carbon atom of the C ring, a double bond between the second and third carbon atoms in this ring, and the number of hydroxyl groups or other groups. All naturally occurring flavonoids have three hydroxyl groups: two in ring A (position 5 and 7) and one in ring B (position 3) [14]. The different position of the substituents in the flavonoid molecule gives it different chemical and physical properties, which translates into the individual metabolism of a given compound and its biological activity. The well-documented biological activity of flavonoids is related to anti-oxidant, -cancer, -inflammatory, -aggregation, -atherosclerotic, -hypertensive and -arrhythmic properties [15]. 

The metabolism of flavonoids is not fully understood. These compounds are ingested with the diet and metabolized mainly in the intestine and liver. Depending on their chemical structure, flavonoids are transported differently from the intestinal lumen to enterocytes. Hydrophobic aglycons are transported by passive diffusion, while flavonoid glycosides (more hydrophilic) are transported by active transport, with the participation of the Na^+^/glucose transporter. The high molecular weight of glycosides reduces their absorption in the small intestine. Only in its further parts are they hydrolyzed with the participation of β-glucosidases to aglycone and sugar, and then absorbed in the large intestine. In enterocytes, the glucuronidation of aglycons occurs with the participation of UDP-glucuronyl transferase, and flavonoid methylation may also take place [16]. After the absorption of aglycone, the heterocyclic backbone is cleaved and the phloroglucinol and phenolic acids are formed. The absorbed end products of flavonoid break down are transported to the bloodstream, where they bind to albumin. Unabsorbed flavonoids are mainly excreted in the urine, and a small amount in the feces [17]. In the liver, flavonoids are demethylated and hydroxylated with the participation of cytochrome P-450 (monooxygenases responsible for the first phase of the detoxification process), and then, due to the activity of second phase detoxification enzymes, primary flavonoid metabolites are O-methylated and/or conjugated with sulfuric and glucuronic acids, and subsequently transported with blood to the tissues, where they show different biological activity. The flavonoids consumed with the diet are not accumulated in the body, and some of their metabolites formed in the liver are excreted into the bile entering the enterohepatic circulation, where they are reabsorbed from the intestine, and the rest are excreted by the kidneys [18]. Modifications of flavonoids during their metabolism may change the activity of these compounds and affect the rate of their removal from the bloodstream. The efficiency of the absorption of individual flavonoids by the body is influenced by the physical and chemical properties of consumed compounds, the type and presence of functional groups, as well as gender, which is probably associated with the increased activity of cytochrome P-450 in men [17]. 

Flavonoids show promising effects in improving cognitive functions. It has been shown that a diet rich in flavonoids negatively correlates with cognitive impairment (OR 0.39; 95% CI 0.15–1.00). Moreover, a correlation was seen between the consumption of flavonoid subclasses (catechins (OR 0.24; 95% CI 0.08–0.72), anthocyanins (OR 0.38; 95% CI 0.14–1.00), flavan-3-ols (OR 0.30; 95 % CI 0.11–0.76) and flavanols (OR 0.30; 95% CI 0.11–0.76) and cognitive function [19]. A flavonoid-rich diet improves cognition regardless of age. In a study evaluating the effects of the consumption of a flavonoid-rich drink (240 g) with wild blueberry on short-term memory, executive functions, reading and mood in children (7–10 years), it was shown that 2 h after consumption supplemented with flavonoids, executive functions improved (attention span, verbal memory), but there was no effect on reading ability [20]. However, a study conducted in young adults (20–30 years) showed that the consumption of flavonoid-rich mixed berries significantly maintained and/or improved memory and focus, and reduced cognitive fatigue that had been observed over time (2, 4 and 6 h of testing). Cognitive functions in the placebo group decreased over time—between 2 h and 6 h (*p* < 0.001) and 4 h and 6 h (*p* < 0.001)—while they did not change in the study group. Thus, it can be concluded that a diet rich in flavonoids supports cognitive executive function, especially in times of fatigue and times requiring greater concentration [21]. In turn, in the elderly (60–70 years old), in whom cognitive impairment is frequently observed, the administration of grape and berry extract (258 mg of flavonoids) significantly improved the exploratory and episodic memory, especially in the subgroup showing the greatest impairment [22].

### 2.1. Flavonols

One sub-class of flavonoids is the flavonols, which include 3-hydroxyflavone backbone (3-hydroxy-2-phenylchromen-4-one). These compounds give flowers a yellow color, and they come in the form of aglycons and B-glycosides. In a given type of fruit, the content of flavonols depends on the variety and the degree of ripeness of the fruit (this also applies to other bioflavonoids) [23].

Icariin (5-hydroxy-2-(4-methoxyphenyl)-8-(3-methylbut-2-enyl)-7-[(2S,3R,4S,5S,6R)-3,4,5-trihydroxy-6-(hydroxymethyl)oxan-2-yl]oxy-3-[(2S,3R,4R,5R,6S)-3,4,5-trihydroxy-6-methyloxan-2yl]oxychromen-4-one, ICA) is an organic inhibitor of phosphodiesterase-5 (PDE5) with phytoestrogen activity, extracted from perennials of the genus *Epimedium*, which penetrates the BBB. ICA has a wide therapeutic effect, including anti-inflammatory and antioxidant effects, and is also used in erectile dysfunction through the activation of the nitric oxide/cyclic guanosine monophosphate (NO/cGMP) pathway [24,25,26]. It was shown that ICA treatment increased levels of cGMP and NO, through a rise in both the activity and gene expression of three isoforms of nitric oxide synthase. The activation of the NO/cGMP pathway by ICA in a mouse model of Alzheimer’s disease (AD) improved memory and learning ability. In the cortex and hippocampus, an elevated level of the following proteins was directly involved in the formation of senile plaques: amyloid precursor protein (APP), amyloid-beta (Aβ1-40/42) and PDE5. After ICA administration (30–60 mg/kg), these molecules’ levels were decreased, both at the mRNA and protein levels [24]. It was also demonstrated that ICA (administered orally at a dose of 3–30 mg/kg) dose-dependently upregulated the expression of BDNF, postsynaptic density protein 95 (PSD-95) and synaptophysin in a traumatic brain injury (TBI) mouse model [25]. Icariin also had neuroprotective effects in cerebral ischemia/reperfusion (I/R) injuries. In animal models, it has been found that ICA prevents neuronal damage while increasing recognition ability. The detailed mechanism of action is not fully understood, but it has been suggested that the neuroprotective effects of ICA may be related to the peroxisome proliferator-activated receptor (PPAR) pathway, which plays an important role in neuroinflammation [27]. Moreover, it has been shown that ICA enhances the viability of neurons and inhibits apoptosis in vitro, by activation of the p38 pathway, improving the activity of antiapoptotic protein sirtuin 1 (SIRT1) and/or downregulating the following proapoptotic proteins: B-cell lymphoma protein 2 (Bcl-2)-associated X (Bax), Becklin-1, protein light 1 chain microtubule-associated 3 (LC3-II) and caspase 3 (CASP3), as well as upregulating the antiapoptotic protein B-cell lymphoma protein 2 (Bcl-2) [28].

In turn, the phytochemicals contained in *Ginkgo biloba*—bilobalide ((1S,4R,7R,8S,9R,11S)-9-tert-butyl-7,9-dihydroxy-3,5,12-trioxatetracyclo[6.6.0.0^1,11^.0^4,8^]tetradecane-2,6,13-trione) and quercetin (2-(3,4-dihydroxyphenyl)-3,5,7-trihydroxychromen-4-one)—exhibited proliferative effects in hippocampal neurons, as well as enhancing synaptogenesis in a dose-dependent manner. These compounds increased the phosphorylation of CREB, as well as the levels of BDNF and pCREB in mice. It has been suggested that both flavonols owe their neurorestorative properties to a common signaling pathway in which CREB is involved [29]. Quercetin is one of the most abundant flavonoids in the natural environment, mainly in the form of *O*-glycoside derivatives, and rarely as aglycone. It is contained in various organs (fruits, shoots, flowers, leaves) in many species of plants, such as vegetables (cabbage, spinach, onions and others), fruits (grape, blackcurrant, blueberry, apples, etc.), flowers (black lilac, horse chestnut, hawthorn), as well as in herbs (St. John’s wort, horsetail, chamomile, etc.), and also in products of plant origin (wine, tea, honey, and others) [30]. The well-known pro-health effect of quercetin is related to its anti-oxidant, -cancer, -allergic, -inflammatory, -platelet, -obesity, -hypertensive, -atherosclerotic and -hypercholesterolemic properties [31]. Studies in pig and rat models have shown that quercetin penetrates the BBB, but its cerebral concentration is very low (from pico- to nanomolar); however, increasing quercetin permeation can be achieved by co-administrating it with α-tocopherol [32]. The neuroprotective effect of quercetin is related to the suppression of neuroinflammation and an antioxidant effect. In vitro studies have shown that quercetin inhibited cellular toxicity, reducing oxidative stress in primary neurons and the neuronal cell line [33]. Moreover, quercetin glycosides (isoquercetin and rutin) showed neuroprotective effects by affecting the expression of anti-apoptotic genes (*Opa1* and *NSF*), as well as upregulating ion transport in the cellular model of Parkinson’s disease (PD) [34]. It should be noted that the observed neurorestorative effects of quercetin in vitro were noticeable at micromolar concentrations, and low concentrations (pico- and nanomolar) were noted in the brains of pigs and rats after in vivo administration [35,36]. Nevertheless, in vivo studies found that administering quercetin to mice that are fed a high-fat diet counteracted cognitive deficits [37], while in the rat model of intracerebral hemorrhage the flavonoid supported behavioral and neuronal regeneration [38]. Quercetin (5–50 mg/kg) reduced the expression of inflammatory markers (tumor necrosis factor TNF-α, and interleukins IL-1β, IL-6, and IL-4), the number of apoptotic cells, the brain water content and the lesion volume in a dose-depended manner [38]. Quercetin has also been shown to improve memory impairment and cognitive function in neurodegenerative diseases, including AD and PD, mainly by inhibiting β-amyloid (Aβ) aggregation and tau protein hyperphosphorylation [34,39,40,41]. Importantly, the oral administration of quercetin (40 mg/kg/day) enhanced neurogenesis in the dentate gyrus of the hippocampus by increasing the expression of genes involved in its regulation (*NGF*, *BDNF*, *EGR-1*, *CREB*) in an AD rat model [39]. In addition, quercetin has been reported to reduce the thinning of all retinal layers caused by I/R injury through its anti-apoptotic effect [42].

### 2.2. Flavones

Flavones containing the 2-phenylchromen-4-one (2-phenyl-1-benzopyran-4-one) backbone are natural yellow dyes. Flavones are present as glycosides in the fruits, leaves and flowers of plants. They are contained in a variety of plants, including citrus fruit, parsley, celery, red pepper, mint, chamomile and more. They have well-defined anti-oxidant, -inflammatory, -carcinogenic and -obesogenic properties [43].

7,8-dihydroxyflavone (7,8-dihydroxy-2-phenylchromen-4-one, 7,8-DHF) is a naturally occurring small molecule agonist of the tropomyosin B receptor (TrkB), a mimetic of BDNF. In vivo studies have shown that 7,8-DHF penetrates the BBB and binds strongly to TrkB, causing its dimerization and autophosphorylation, leading to the activation of further signaling cascades [44]. Animal model studies have shown a beneficial effect of 7,8-DHF in the treatment of numerous diseases of the nervous system, including Rett syndrome [45], PD [46,47], depression [48], Huntington disease [49] and amyotrophic lateral sclerosis (ALS) [50]. It was found that 7,8-DHF orally administered in a mouse AD model restored memory impairment, reduced β-secretase (BACE1) (the protein responsible for APP proteolysis), increased Aβ level, and improved TrkB signaling [51,52,53]. Moreover, 7,8-DHF, by activating the TrkB pathway, promoted dendritic branching, the survival of cortical neurons, and synaptogenesis. The oral administration of 7,8-DHF (5 mg/kg/day) in a mouse AD model inhibited synapse loss in the hippocampus and their plasticity [54]. 7,8-DHF has also shown beneficial effects in studies on age-related memory loss due to the weakening of amygdalar synaptic plasticity in a rat model. Treatment with 7,8-DHF, administered intraperitoneally at a dose of 5 mg/kg/day, enhanced dendritic spine density and number in the amygdala, hippocampus and prefrontal cortex, increased phosphorylated TrkB activation, and enabled the synaptic plasticity of basolateral amygdala [55].

Baicalein (5,6,7-trihydroxy-2-phenylchromen-4-one), and its 7-*O*-glucuronide baicalin ((2S,3S,4S,5R,6S)-6-(5,6-dihydroxy-4-oxo-2-phenylchromen-7-yl)oxy-3,4,5-trihydroxyoxane-2-carboxylic acid), are flavonoids extracted from *Scutellaria*, widely used in Chinese medicine as anti-inflammatory, -viral, -bacterial, -apoptotic -oxidant and -coagulant substances [56]. The administration of baicalein and baicalin to rats with induced stroke reduced the infarct volume, and improved motor, cognitive and behavioral skills, and neurological deficit [57,58,59,60,61]. The neuroprotective effects of these flavonoids were related to the inhibition of CaMKII phosphorylation, as well as the modulation of mitochondrial function. Moreover, baicalin supplementation (50–100 mg/kg for 7 days) has been shown to increase synaptic plasticity in a dose-depended manner in the hippocampus of mice with I/R injury [59]. Another study suggested that the neurorestorative properties of baicalin may be associated with toll-like receptor 2 and 4 (TLR 2/4) pathways. In the rat brain, baicalin has been shown to reduce the nuclear factor ’kappa-light-chain-enhancer’ of activated B-cells (NF-κB) and TLR 2/4 expression, as well as both the activity and expression of COX-2 and iNOS, and the serum levels of IL-1β and TNF-α [58,60,61]. What is more, baicalein, through the BDNF/CREB pathway, and by inhibiting oxidative stress, reduced cognitive deficits and neurogenesis disorder in the hippocampus of mice exposed to γ-ray radiation [62]. The neurogenic activity of baicalein (50 mg/kg, administered orally) was also associated with the regulation of glycogen synthase kinase 3 (GSK3b), angiopoietin 1 (Ang-1) and Akt levels in neuronal cells. The phosphorylation of GSK3b by Akt inhibits neuronal apoptosis and promotes the proliferation of nerve cells [63]. In turn, a study using a mouse PD model showed that baicalein, through the upregulation of gene expression (mainly glutamate receptor, *GLR*, alpha-synuclein, *SNCA*, and LIM domain kinase 1, *LIMK1*) promoted not only neurogenesis, but also neurotrophin pathway signaling, neuroblast proliferation, and motor and behavioral improvement [64].

### 2.3. Flavan-3-ol

Flavan-3-ols are flavans derivatives with a 2-phenyl-3,4-dihydro-2H-chromen-3-ol backbone. Depending on the modification within the heterocyclic ring, the following free form compounds are distinguished: (+)-catechin, (−)-epicatechin (EC), (+)-galocatechin (GC), (−)-epigallocatechin (EGC). So too are the following bound catechins: (−)-galocatechin gallate (GCG), (−)-epicatechin gallate (ECG) and (−)-epigallocatechin gallate (EGCG). They have anti-oxidant, -mutagenic, -diabetic and -cancer activities [65].

Epigallocatechin gallate ([(2R,3R)-5,7-dihydroxy-2-(3,4,5-trihydroxyphenyl)-3,4-dihydro-*2H*-chromen-3-yl]3,4,5-trihydroxybenzoate), found in significant amounts in green tea, is a powerful antioxidant with a suppressive and anti-inflammatory effect. Moreover, EGCG exhibits a neuroprotective activity that has been proven in stroke [66], AD, PD [67], and spinal cord injuries [68]. EGCG, by inhibiting the TLR/NF-κB pathway, has been shown to reduce IL-1β, TNFα and IL-6 levels, promoting lipopolysaccharide-impaired neurogenesis [69], and improving epilepsy-induced cognitive function and synaptic dysfunction [70]. Other studies suggest that the neuroprotective effects of EGCG are associated with the activation of CREB/BDNF/TrkB-PI3K/Akt signaling. It has been shown that after EGCG administration, there is an increase in the levels of Akt, mTORc1, phospho-Akt and phospho-GSK3b, as well as an increase in *BDNF* and *TrkB* mRNA expression. Thus, EGCG affects the preservation of memory, improves learning ability, and enhances neuronal survival [71]. In addition, EGCG (2.5 mg/kg for 2 weeks) in the dentate gyrus has been shown to promote the proliferation of neuronal precursor cells as well as inhibit apoptosis in the hippocampus [72]. Moreover, EGCG (administered intraperitoneally, at a dose of 20 mg/kg) in rat models with I/R injury reduced neurological deficits, decreased oxidative stress and levels of brain injury markers, promoted neuron survival, and inhibited the apoptosis of neuronal cells through the upregulation of Bcl-2 expression and the downregulation of Bax and Caspase-3 [66].

Similarly, a high consumption of cocoa, rich in flavan-3-ols, including (−)epicatechin ((2R,3R)-2-(3,4-dihydroxyphenyl)-3,4-dihydro-2H-chromene-3,5,7-triol), resulted in increased cortical blood flow as well as vascular function, especially in the hippocampus. Van Praag et al. investigated the effect of a diet enriched with (−)epicatechin (at a level of 500 μg/g) on brain function, in particular on the improvement of cognitive functions in C57BL/6 mice. (−)Epicatechin improved spatial memory in mice, with simultaneous (i) upregulation of gene expression related to angiogenesis and learning (Nuclear factor of activated T-cells, cytoplasmic, calcineurin-dependent 1, *Nfatc1*; Squamous cell carcinoma antigen recognized by T-cell 2, *Sart2*; Notch gene homolog 1, *Notch1*; Protein tyrosine phosphatase, nonreceptor type 6, *Ptpn6*; Parvin γ, *Parvg*; Microfibrillar-associated protein 2, *Mfap2*; Zinc finger protein 553, *Zfp553*; Cell division cycle 20 homolog, *Cdc20*; MAD homolog 5, *Smad5*; and Protein-O-mannosyltransferase 2, *Pomt2*), as well as (ii) downregulation of the following apoptotic and inflammatory genes: Chloride channel calcium activated 5, *Clca5*; Exosome component 2, *Exosc2*; CD52 antigen, *Cd52*; Scleraxis, *Scx*; Transmembrane 4 superfamily member 5, *Tm4sf5*; Seminal vesicle secretion 6, *Svs6*; Tripartite motif-containing 45, *Trim45*; Lipoprotein lipase, *Lpl*; Ribosomal protein L35, *Rpl35*; and Hairy and enhancer of split 3, *Hes3* [73].

### 2.4. Isoflavones

Isoflavones are phytohormones, mimetics of estrogens. Isoflavones differ from other flavonoid compounds by way of a modified ring structure. Their characteristic feature is the presence of the B ring at the C3 position, instead of the C2 position, and the formation of a 3-phenylpropane backbone. The chemical structure of isoflavones is similar to that of 17-β-estradiol, meaning they have an affinity for estrogen receptors, mainly ERβ, and to a lesser extent also for ERα. The sources of isoflavones include soybeans, lentils, red clover and spinach [74].

Biochanin A (7-dihydroxy-3-(4-methoxyphenyl)chromen-4-one, BCA) is an isoflavone, a methylated precursor of genistein, which penetrates the BBB. Its sources can be red clover, alfalfa, cabbage and others, and it is often used in supplements intended for perimenopausal women. It has estrogen-like, anti-inflammatory, anti-cancer, anti-diabetic and lipid metabolism-regulating properties [75]. It has been shown that BCA reduces neurological deficits in an I/R injury rat model [76,77]. It has been noted that 14-day BCA supplementation, at a dose of 10–40 mg/kg, resulted in a dose-depended reduction in the volume of stroke and cerebral edema in rats, with a simultaneous reduction in the parameters of neuroinflammation (levels of IL-1β and TNF-α) and myeloperoxidase (MPO) activity by inhibiting the p38 pathway [77]. In turn, the inhibition of the TLRs/TIRAP/MyD88 /NF-κB pathway caused by BCA improved neurological deficits and reduced neuroinflammation in a rat model of brain injury induced by subarachnoid hemorrhage [78]. BCA (5–10 mg/kg) caused a dose-dependent upregulation of both the gene and protein expression of glutamate oxaloacetate transaminase (GOT) in neurons, leading to the inhibition of glutamate-induced apoptosis in a mouse model of stroke [76]. Moreover, BCA has shown a neuroprotective effect against the LPS-damage of dopaminergic neurons by inhibiting the MAPK pathway in the microglia [79]. Its neuroprotective effect is also associated with antioxidant activity. As shown in studies in PD rat models, BCA increased the survival of neurons by enhancing the activity of superoxide dismutase (SOD) and glutathione peroxidase (GPx), and reducing the production of malondialdehyde (MDA) in the brain, and therefore reducing neuroinflammation [80,81].

Equol is a flavonoid formed by the conversion of daidzein to dihydrodaidzein, induced by intestinal bacteria. Equol has a very strong estrogenic effect, but its synthesis takes place only in some people. This compound can exist in two forms, known as the *S*-(−)equol ((3S)-3-(4-hydroxyphenyl)-3,4-dihydro-2H-chromen-7-ol) and R-(−)equol diastereoisomers. However, only *S*-equol is produced in the digestive systems of many different species of animals (monkeys, rats, mice, chimpanzees, cows, pigs and hens), as well as in about 30–50% of people after soy product consumption [82]. In recent years, *S*-equol has raised more and more clinical hopes, and research suggests that it may have neuroprotective potential. This compound has been shown to antagonize Aβ-induced neuronal apoptosis in SH-SY5Y neuroblastoma cells. Moreover, *S*-equol reversed the downregulation of Erα and steroid receptor coactivator-1 (SRC-1) expression, and ERK 1/2 activation, thus inhibiting cytotoxicity caused by Aβ, and promoted neuronal survive [83]. Moreover, in vitro studies showed that in LPS-activated microglia cells, equol inhibited the activity of MAPK, TLR and NF-kB, the secretion of IL-6, TNF-α and prostaglandin E2 (PGE-2), and nitric oxide production. Moreover, equol inhibited neuronal apoptosis, and enhanced NGF production and neurite outgrowth [84]. Equol also has neuroprotective potential in ischemic brain injuries [85,86,87]. It has been shown that in stroke-induced rats, equol, administered intragastrically at a dose of 0.625–2.5 mg/kg, reduced neurological deficit, the mortality of animals, as well as the volume of ischemia. Moreover, equol reduced the levels of phosphorylated Src-tyr416 and gp91, which mediate ischemic neuronal damage [86]. In addition, in vitro studies have shown that this metabolite dose-dependently decreased the level of MDA and the activity of lactate dehydrogenase, thereby increasing the viability of nerve cells [87].

### 2.5. Anthocyanins

Anthocyanins are glycosides containing the aglycone part of an anthocyanidin skeleton, composed of two phenyl rings connected by a three-carbon aliphatic chain. In plants, they occur in the form of polyhydroxy and polymethoxy glycoside derivatives of the flavylic cation 2-phenylbenzopyryl. These compounds are widely found in many plants, except algae and plants from the cactus and quinoids families. They are found mainly in flowers, fruits and seeds, but also in plant leaves. They are natural plant dyes ranging in color from red to dark purple, the color depending on the environmental pH and chelation by metal ions. They have well-documented anti-oxidant, -inflammatory, -carcinogenic, -obesogenic, -diabetic, -hypertensive and -edema properties [88]. Importantly, it has been shown that anthocyanins are absorbed from the gastrointestinal tract in humans, are able to penetrate the BBB, and are located in various areas of the brain, such as in the hippocampus, cerebellum, striatum and cortex [89]. Studies on an animal model have shown that supplementation with blueberry (2% *w/w*) for 12 weeks, containing 420 μg anthocyanins/g feed (delphinidin, 98.2 μg/g feed; cyanidin, 18.0 μg/g feed; petunidin, 42.6 μg/g feed; peonidin, 3.8 μg/g feed; malvidin, 104.6 μg/g feed), significantly improved memory in older rats. Memory improvement positively correlated with an increase in BDNF level and CREB activation in the hippocampus. Moreover, an increased activation of the ERK 1/2 and Akt signaling pathways and mTOR activation in the hippocampus have been demonstrated. There was also an increase in the level of activity-regulated cytoskeleton-associated protein (Arc/Arg3.1), an early marker of synaptic stimulation [90].

Cyanidin (2-(3,4-Dihydroxyphenyl)chromenylium-3,5,7-triol, Cy) is an orange-red vegetable pigment, which, depending on the pH of the environment, can change its color (pH < 3—red, 8–9—blue, and <11—purple). It is used in the food industry as a red dye with the number E163a. It is found in red fruits, including berries, grapes, lilac, hawthorn, hibiscus flower, red onion and red cabbage. It has strong anti-oxidant, -cancer, -inflammatory and -diabetic properties [91]. It has been shown that Cy is capable of penetrating the BBB, and the administration of Cy (50 mg/kg) promotes neurogenesis in the mouse hippocampus via the PI3K/AKT/FOXG1/FGF-2 pathway. Cy increased the number of doublecortin cells (a marker of immature neurons) and tertiary dendrites, the dendritic length, as well as the levels of 5-HT and NE [92]. In turn, in mice with I/R injury, the administration of purified anthocyanin extract from *Myrica rubra*, which contained 21.28% cyanidin-3-*O*-glucoside, at a dose of 100–300 mg/kg, resulted in a dose-dependent reduction in the infarct volume, and also had a neuroprotective effect via the TLR4/NF-κB and NOD-like receptor pyrin domain-containing 3 protein (NLRP3) pathways. The Nod-like receptor protein-3 (NLRP3) inflammasome is composed of NOD-like receptor 3, procaspase 1, and the adaptor protein apoptosis-associated speck-like protein, comprising a caspase recruitment domain (ASC). Inhibition of the activity of the large molecule multiprotein complex of the inflammasome, consisting of NLRP3, has great therapeutic potential due to its participation in a strong inflammatory response in the CNS [93]. The neuroprotective effect of Cy is also associated with the anti-apoptotic effect through the Akt and ERK 1/2 pathways [94].

Malvidin (3,5,7-trihydroxy-2-(4-hydroxy-3,5-dimethoxyphenyl)chromenium, Mv) is an organic compound from the group of anthocyanides, used in the food industry as a violet dye with the number E163C. The main source of Mv is *Vitis vinifera*, but it is also present in the fruits and flowers of many plants, including blueberries and cranberries. Mv and its 3-*O*-β-glucoside are characterized by strong anti-inflammatory properties, enacted by reducing the transcription of genes encoding the pro-inflammatory mediators TNFα, interleukins 1 and 6. Moreover, Mv has been shown to reduce the production of NO in macrophages without the reported toxic effect [95]. It has been found that the oral administration of Mv-3-*O*-glucoside (0.5 µg/kg) regulates synaptic plasticity by increasing histone acetylation in Rac1 gene regulatory sequences [96]. It has also been reported that the phytochemicals from the maqui berry, administered in a ration (25–100 mg/kg), including Mv-3-*O*-glucoside, showed a dose-dependent antidepressant effect associated with the inhibition of oxidative stress in post-stroke mice [97]. Furthermore, anthocyanins extracted from *Ribes meyeri* have increased the proliferation and survival of murine NSCs, demonstrating an anti-aging effect. The anthocyanin-treated NSCs have shown decreased levels of oxidative markers, a greater number of cells in the S phase, lower expressions of the aging gene p16^ink4a^, and extended telomeres. In addition, mice in the study group showed improved cognitive function, but with no differences in motor function, compared to the control group [98].

### 2.6. Other Flavonoids

Resveratrol (5-[(E)-2-(4-hydroxyphenyl)ethenyl]benzene-1,3-diol) is a natural polyphenolic phytoalexin found in abundance in numerous plants, including berries, grapes and nuts. There are studies that indicate the strong anti-inflammatory, -aging, -apoptotic, -oxidant, -diabetic and -cancer, as well as cardioprotective and hepatoprotective, properties of resveratrol. It has been proven to be helpful in the treatment of various diseases, including cancer, cardiovascular diseases and diabetic retinopathy [99]. As shown in in vitro and in vivo studies, resveratrol inhibits axonal degeneration after injury, and promotes neurite outgrowth and synaptogenesis in primary neurons, Neuro2a cells, AD neurons and sensory neurons [100,101,102]. Moreover, after oxygen–glucose deprivation/reoxygenation injury in vitro, resveratrol activated the sonic hedgehog homolog (Shh) pathway leading to the activation of Sirt1, and thus enhanced synaptogenesis and neurite growth [103]. Furthermore, the neuroprotective effects of resveratrol were associated with the activation of the nuclear erythroid 2-related factor 2 (Nrf2)/HO-1 pathway by increasing the expression and activity of SOD, catalase (CAT) and GPx, and reducing the MDA level in brain tissue. Importantly, it improved the anti-oxidative parameters correlated with improved spatial memory in a mouse model of AD disease. In addition, resveratrol reduced the expressions of Aβ and ERβ, as well as increasing the expressions of choline acetyltransferase (ChAT), estradiol, and both the protein and mRNA of Erα [104]. Significantly, it is suggested that female sex hormones positively correlate with the improvement of cognitive functions and memory, and the increased secretion of neurotransmitters indirectly affects synaptogenesis [105,106,107]. Another study in vitro showed that the inhibition of Aβ aggregation by resveratrol (100 µM) was achieved by regulating the following proteins involved in proteostasis: ubiquitin-like protein (UBL) and X-box 1 binding protein (XBP-1) [108]. The neuroprotective effect of resveratrol administration, enacted by activating the Nrf2 pathway and thus inhibiting neuroinflammation, apoptosis and oxidative stress, has also been noted in other CNS diseases, such as vascular dementia [109], stroke [110], spinocerebellar ataxia type 3 [111], and traumatic brain injury [112]. In contrast, in the PD rat model, resveratrol (20 mg/kg) inhibited cerebral CASP3 activity, while downregulating the expression of the glucose-regulated protein 78 (*GRP78*) and C/EBP homologous protein (*CHOP*) genes, resulting in a reduction in endoplasmic reticulum stress-induced apoptosis [113]. Moreover, this flavonoid increased the expression of miR-214, which resulted in a decrease in α-synuclein mRNA expression, which is a presynaptic neuronal protein neuropathologically and genetically related to PD and functional improvement in animals [114].

Numerous studies on the health-promoting effects of a plant from the celery family, *Centella asiatica*, indicate its neuroplastic effect as dependent on the content of phytochemicals phenylpropanoid derivatives (flavonoids, caffeoylquinic acids, and eugenol derivatives) and isoprenoids (pentacyclic triterpenoids, sesquoniterins and sterols) [115]. The neurotrophic effect of *C. asiatica* is related to the regulation of the Akt and ERK1/2 signaling pathways leading to dendritic synaptogenesis and arborization [116], while the neuroprotective effect is ascribed to increased mitochondrial activity, inhibition of phospholipase A2 [117] and anti-oxidant properties [118]. Among all the phytochemicals contained in *C. asiatica*, the most prominent neuroprotective and neurotrophic importance is attributed to flavonoids, which increase the Nrf2-antioxidant response pathway [115].

## 3. Conclusions

The complex etiology of CNS diseases is a serious therapeutic problem. Compounds enhancing endogenous neuroplasticity raise high hopes in CNS therapy. The direct administration of neurotrophins often does not bring the expected result. On the other hand, compounds of natural origin, including flavonoids, actively support neuroplasticity, have neuroprotective effects, and are characterized by low toxicity (Table 1). The neurorestorative actions of flavonoids are associated with both antioxidant and anti-inflammatory properties, but also act through the activation of multiple pathways responsible for synaptogenesis and neurogenesis. Thus, the inclusion of flavonoids in the treatment of both neurodegenerative and ischemic diseases has great therapeutic potential.

## Figures and Tables

**Table 1:** Summary of neuroprotective effects of individual representatives of the main flavonoid groups.

Flavonoid	Iupac Name	Role	Signaling Pathway
**Flavonols**			
Icariin	5-hydroxy-2-(4-methoxyphenyl)-8-(3-methylbut-2-enyl)-7-[(2S,3R,4S,5S,6R)-3,4,5-trihydroxy-6-(hydroxymethyl)oxan-2-yl]oxy-3-[(2S,3R,4R,5R,6S)-3,4,5-trihydroxy-6-methyloxan-2yl]oxychromen-4-one	Improvement of memory and learning ability Synaptogenesis Neuronal survival Neuroprotection Inhibition of apoptosis	NO/cGMP pathway [24,25,26] CREB/BDNF/TrkB-PI3K/Akt pathway [25] p38 pathway [28]
Bilobalide	(1S,4R,7R,8S,9R,11S)-9-tert-butyl-7,9-dihydroxy-3,5,12-trioxatetracyclo[6.6.0.01,11.04,8]tetradecane-2,6,13-trione	Neuronal proliferation Synaptogenesis	CREB/BDNF/TrkB-PI3K/Akt pathway [29]
Quercetin	2-(3,4-dihydroxyphenyl)-3,5,7-trihydroxychromen-4-one	Neuronal proliferation Synaptogenesis Improvement of memory and learning ability Inhibition of apoptosis Neuroprotection	CREB/BDNF/TrkB-PI3K/Akt pathway [29]
**Flavones**			
7,8-dihydroxyflavone	7,8-dihydroxy-2-phenylchromen-4-one	Dendritic branching Survival of cortical neurons Synaptogenesis	CREB/BDNF/TrkB-PI3K/Akt pathway [55]
Baicalein Baicalin	5,6,7-trihydroxy-2-phenylchromen-4-one (2S,3S,4S,5R,6S)-6-(5,6-dihydroxy-4-oxo-2-phenylchromen-7-yl)oxy-3,4,5-trihydroxyoxane-2-carboxylic acid	Reduction of the infarct volume Improvement of motor, cognitive and behavioral skills Reduction of neurological deficit	Inhibition of CaMKII phosphorylation [59] TLR 2/4 pathway [60] P3K/Akt pathway [63]
**Flavan-3-ols**			
Epigallocatechin gallate	([(2R,3R)-5,7-dihydroxy-2-(3,4,5-trihydroxyphenyl)-3,4-dihydro-2H-chromen-3-yl]3,4,5-trihydroxybenzoate	Neurogenesis Improvement of cognitive function and synaptic dysfunction Neuronal survival Apoptosis inhibition Neuronal proliferation	TLR/NF-κB pathway [69] CREB/BDNF/TrkB-PI3K/Akt pathway [71]
(-)epicatechin	(2R,3R)-2-(3,4-dihydroxyphenyl)-3,4-dihydro-2H-chromene-3,5,7-triol	Improvement of cognitive functions	CREB/BDNF/TrkB-PI3K/Akt pathway [73]
**Isoflavones**			
Biochanin A	7-dihydroxy-3-(4-methoxyphenyl)chromen-4-one	Reduction of neurological deficit Apoptosis inhibition Neuroprotection	p38 pathway [77] TLRs/TIRAP/MyD88 /NF-κB pathway [78] MAPK pathway [79]
*S*-equol	(3S)-3-(4-hydroxyphenyl)-3,4-dihydro-2H-chromen-7-ol	Synaptogenesis Neuronal survival Neuroprotection Inhibition of apoptosis	ERK 1/2 pathway [84]
**Anthocyanins**			
Cyanidin	2-(3,4-Dihydroxyphenyl)chromenylium-3,5,7-triol	Neurogenesis Neuroprotection Inhibition of apoptosis	PI3K/AKT/FOXG1/FGF-2 pathway [92] TLR4/NF-κB pathway [93] NLRP3 pathways [93] Akt and Erk 1/2 pathways [94]
Malvidin	3,5,7-trihydroxy-2-(4-hydroxy-3,5-dimethoxyphenyl)chromenium	Neuroprotection Inhibition of apoptosis Synaptic plasticity	Histone acetylation in Rac1 [96] Erk 1/2 pathway [98]
Resveratrol	5-[(E)-2-(4-hydroxyphenyl)ethenyl]benzene-1,3-diol	Inhibit of axonal degeneration after injury Promotion of neurite outgrowth Synaptogenesis	Shh pathway [103] (Nrf2)/HO-1 pathway [104] Nrf2 pathway [109,110,111,112]
